# Development of a bacterial consortium from *Variovorax paradoxus* and *Pseudomonas veronii* isolates applicable in the removal of BTEX

**DOI:** 10.1186/s13568-022-01349-2

**Published:** 2022-01-25

**Authors:** Flóra Szentgyörgyi, Tibor Benedek, Dzsenifer Fekete, András Táncsics, Péter Harkai, Balázs Kriszt

**Affiliations:** 1 Department of Molecular Ecology, Institute of Aquaculture and Environmental Safety, Hungarian University of Agriculture and Life Sciences, Gödöllő, Páter K. u. 1, 2100 Hungary; 2 Soós Ernő Research and Development Center, Circular Economy University Center, University of Pannonia, Nagykanizsa, Zrínyi u. 18, 8800 Hungary; 3 Department of Environmental Safety, Institute of Aquaculture and Environmental Safety, Hungarian University of Agriculture and Life Sciences, Gödöllő, Páter K. u. 1, 2100 Hungary

**Keywords:** *Variovorax*, *Pseudomonas*, Bacterial consortium, Bioaugmentation, BTEX biodegradation

## Abstract

In this study, we report on the development of a novel bacterial consortium, consisting of *Variovorax paradoxus* and *Pseudomonas veronii* isolates, applicable in the biodegradation of all six BTEX compounds (benzene, toluene, ethylbenzene, *o*-, *m*- and *p*-xylene) and the bioremediation of contaminated sites. The co-cultivability of the selected bacterial isolates was determined in nutrient-rich medium, as well as in BTEX amended mineral salts solution using Terminal Restriction Fragment Length Polymorphism (T-RFLP) and CFU determinations. BTEX biodegradation capacity of the two-strain consortium was assessed in mineral salts solution, where a series of BTEX depletions and supplementations occurred, as well as in a real, BTEX polluted environmental sample (contaminated groundwater) in the presence of the autochthonous bacterial community. The obtained results indicated that the developed bacterial consortium is very efficient in BTEX biodegradation. Under laboratory conditions, the acclimatized bacterial consortium completely degraded the BTEX mixture with a concentration as high as 20 mg l^−1^ in a mineral salt medium within a short span of 6 h. Close to in situ groundwater conditions (incubated at 15 °C under static conditions in the absence of light), groundwater microcosms containing the autochthonous bacterial community inoculated with the developed bacterial consortium showed more efficient toluene, *o*-, *m*-and *p*-xylene biodegradation capacity than microcosms containing solely the native microbial population originally found in the groundwater. In the inoculated microcosms, after 115 h of incubation the concentration (~ 1.7 mg l^−1^ each) of *o*-, *m*- and *p*-xylene decreased to zero, whereas in the non-inoculated microcosms the concentration of xylene isomers was still 0.2, 0.3 and 0.3 mg l^−1^, respectively. The allochthonous bioaugmentation of the contaminated groundwater with the obtained inoculant was successful and manifested in a better BTEX degradation rate. Our results suggest that the obtained bacterial consortium can be a new, stable and efficient bioremediation agent applicable in the synergistic elimination of BTEX compounds from contaminated sites.

**Supplementary Information:**

The online version contains supplementary material available at 10.1186/s13568-022-01349-2.

## Key points

- Synergistic BTEX metabolism by a *Variovorax* and *Pseudomonas* co-culture
- Complete BTEX biodegradation (20 mg l^−1^) within only 6 h
- Efficient BTEX biodegradation in the presence of the autochthonous microbial community.

## Introduction

According to the U.S. EPA, BTEX compounds (benzene, toluene, ethylbenzene, *o*-, *m*- and *p*-xylene) are considered as priority pollutants with significant public health concerns. They are included in the list of Hazardous Air Pollutants (rank 78) in the CERCLA List from the 275 substances determined as significant threats to human health (Rahul and Balomajumder, 2013). Benzene and ethylbenzene may induce cancer and neurological effects (Smith et al. 2016). Acute exposure to toluene, *o*-, *m*- and *p*-xylene may cause neurotoxicity and reproductive dysfunction (Wilbur and Bosch 2004). Despite their detrimental effects on human health, BTEX are one of the most commonly used chemicals in industries such as petroleum, solvent, paint, adhesives, rubber and pesticide (Abumaizar et al. 1998; Atlas and Philp 2005; Fayemiwo et al. 2017). As a matter of fact, the principal sources of BTEX pollution are the aforementioned industries supplemented with coal and biomass burning or the exhaust emissions in urban environments (Kelley et al. 1997; Baltrenas et al. 2011; Mitra and Roy 2011; Datta et al. 2013; Prestes de Castro et al. 2015; Adamović et al. 2018; Zhang et al. 2020).

BTEX pollutions threaten the pedosphere, hydrosphere and the atmosphere (Máthé et al. 2012; Benedek et al. 2016; Farkas et al. 2017). They are one of the most common water resource and potable water contaminants (Li et al. 2017). In some African and South-East Asian regions, groundwater contamination with BTEX is particularly worrying since the drinking water used for human consumption is extracted from wells and boreholes fed by the groundwater (Fayemiwo et al. 2017). Recently it has been demonstrated that cigarette butt leachate may be a significant source of pollution of aquatic systems with BTEX compounds (Dobaradaran et al. 2021). In an urban atmosphere BTEX are the most abundant volatile organic compounds (VOCs) (Duan and Li 2017; Dehghani et al. 2018).

Based on the above, the minimization of BTEX pollutions and at the same time, the restoration of already polluted sites is crucial from a human and environmental health perspective. In recent decades several physico-chemical remediation techniques have been developed and applied to remove toxic pollutants from the environment, including BTEX. Such techniques include innovative approaches such as advanced oxidation processes, photocatalysis, sonolysis, radiolysis etc. (Mascolo et al. 2008; Braeutigam et al. 2009; Laokiat et al. 2012; Lee et al. 2013; Al-Sabahi et al. 2017; Dhivakar and Rajan 2018; Mohan et al. 2020). However, although these techniques are effective, they usually have a large ecological footprint and are expensive and impractical on a large scale. Biodegradation and bioremediation of BTEX polluted sites would be the greenest and the cheapest way of problem solving if time is not a constraint (Atlas and Philp 2005). Although it has been used for decades, bioremediation is still a popular technique carrying innovative features (Singh et al. 2017; Lee et al. 2019). Bioaugmentation, a type of bioremediation, is the addition of pregrown bacterial cultures to the polluted sites to reduce clean up time and cost (Mrozik and Piotrowska-Seget 2010). It has to be emphasized, that for the most efficient elimination of xenobiotic compounds from the environment a combination of physico-chemical and biological approaches is the most promising. For instance, bioremediation of soils contaminated with high concentrations of crude oil is usually hampered by high toxicity thresholds for microbial degraders (Vasilyeva et al. 2020). In such cases the initial concentration of toxic pollutants can be decreased using physical and/or chemical methods (e.g., adsorption on granulated activated carbon or diatomite; chemical oxidation) and the residues can be easily removed using biological approaches (Alvarez and Illman 2006; Kulik et al. 2006; Vasilyeva et al. 2020).

The task of researchers in the field of bioremediation should be to search for novel and more efficient microbial capabilities for pollutant elimination. Therefore, in this study, we aimed at obtaining a novel bacterial consortium, made up of a *Variovorax* (*Gammaproteobacteria*, *Betaproteobacteriales*) and a *Pseudomonas* (*Gammaproteobacteria*, *Pseudomonadales*) isolate, applicable in bioaugmentation for rapid and complete bioremediation of BTEX-contaminated sites. To the best of our knowledge, in the literature, there is barely any consortium consisting of *Variovorax* and *Pseudomonas* species developed and utilized for the synergistic biodegradation of any kind of organic pollutant. During the study, the BTEX removal capacity of the isolates was tested in the case of single-strains and in co-cultures using either individual compounds or a mixture of BTEX. The co-cultivability and compatibility of strains were tested in nutrient rich media, as well as in mineral salts solution supplemented (spiked) with BTEX as the sole carbon and energy source. The change in the relative abundance of strains during the co-cultivation experiments was determined using cultivation-dependent microbiological and independent molecular biological techniques such as colony-forming unit (CFU) determination and *16S rRNA* gene based terminal restriction fragment length polymorphism (T-RFLP) based on community DNA of co-culture samples, respectively. BTEX biodegradation capacity of the consortium was tested on a real BTEX-contaminated groundwater containing the autochthonous microbial community; allochthonous bioaugmentation of the contaminated groundwater was conducted.

## Materials and methods

### Brief characterization of isolates

*Variovorax paradoxus* strain BFB1_13 was previously isolated from a bacterial biofilm community selectively enriched on BTEX compounds as the sole source of carbon and energy (50 mg l^−1^, the ratio of individual BTEX compounds was 1:1). Enrichments were conducted under aerobic conditions in vitamins and trace elements amended mineral salts solution. When grown on R2A agar plate, strain BFB1_13 forms large, yellowish colonies with a more accentuated yellow color in the middle. Colonies are irregular in shape with raised elevation having high swarming ability (Additional file 1: Fig. S2). As determined previously, isolate BFB1_13 is able to degrade all six BTEX, but mostly benzene, toluene and *o*-xylene, under both aerobic and oxygen-limited conditions (Benedek et al. 2021). Besides I.2.A catechol 2, 3-dioxygenase gene (*C23O*) involved in aerobic BTEX biodegradation, it also harbors at least four different I.2.C subfamily affiliating *C23O* genes, possibly playing a role in the oxygen-limited degradation of simple aromatic hydrocarbons (Kukor and Olsen 1996). Based on the whole genome shotgun sequencing of strain BFB1_13, it is a toolbox of catabolic genes involved in simple aromatic hydrocarbon biodegradation (whole genome sequence GenBank accession number JAEVYQ000000000).

*Pseudomonas veronii* strain BFHA4_7 was isolated from the same biofilm community under similar conditions as strain BFB1_13, but during the enrichment, oxygen-limited conditions were applied; the concentration of O_2_ was set to 0.5 mg l^−1^. Strain BFHA4_7 forms large, shiny and smooth, slightly raised, cream-colored colonies with irregular margins when grown on R2A agar (Additional file 1: Fig. S2). As it was determined previously, it is capable of degrading toluene, *m*- and *p*-xylene. It harbors both I.2.A and I.2.C *C23O*s involved in aerobic and oxygen-limited degradation of simple aromatic compounds, respectively (Benedek et al. 2018). The *16S rRNA* and *C23O* gene sequences can be accessed under the accession numbers MG897142, MW922843 (I.2.A *C23O*) and MG926653 (I.2.C *C23O*).

Originally, the biofilm serving as a source of isolation of the studied isolates developed in a BTEX-contaminated oxygen-limited groundwater on the surface of a stainless-steel submersible pump belonging to a Pump and Treat system (P&T) treating gasoline contaminated groundwater (Benedek et al. 2016 and 2018). Briefly, in 2007 at the contaminated site ∼27 m^3^ of gasoline was leaked accidentally due to the rupture of a refined product pipeline in Central Region of Hungary. 200 tons of soil and the underlying shallow groundwater was contaminated with aliphatic and monoaromatic (BTEX) hydrocarbons. To decontaminate in situ the affected groundwater a physico-chemical P&T system was established within the contamination plume, treating 30 m^3^ contaminated groundwater per day. The still in use P&T system consists of 11 extraction (pumping) and 54 monitoring wells, a central treatment facility, interceptor trenches upgradient and downgradient of the contamination plume, a biofilter, and the associated network of pipes. Massive biofilm formation, the source of isolation of the investigated bacterial strains, occurs on the stainless-steel surface of extraction pumps.

### Determination of BTEX biodegradation capacity of strains

By using either individual or a mixture of compounds, the BTEX biodegradation potential of *V. paradoxus* strain BFB1_13 was thoroughly tested previously under aerobic and oxygen-limited conditions and for planktonic and macro-encapsulated cultures (Benedek et al. 2018, 2021). According to Benedek et al. (2021), *V. paradoxus* strain BFB1_13 completely degraded the mixture of BTEX (conc. ~ 1.3 mg l^−1^ each) within 168 h of incubation under aerobic and oxygen-limited conditions. BTEX degradation by the strain occurred in the following order benzene > toluene > *o*-xylene > ethylbenzene > *m*-, *p*-xylene. On the other hand, as previously determined by Benedek et al. (2018), *P. veronii* strain BFHA4_7 was capable of degrading only toluene, *m*- and *p*-xylene as tested in the presence of individual BTEX compounds (5 mg l^−1^) (Benedek et al. 2018).

#### Determination of BTEX mixture biodegradation capacity of *P. veronii* BFHA4_7

As the first step in this study, the BTEX mixture biodegradation capacity of *P. veronii* strain BFHA4_7 was tested by using a mixture of BTEX compounds according to the following:

In 100 ml hermetically closed crimp sealed serum bottles 50 ml of mineral salts solution (composition CaCl_2_ · 2H_2_O 0.002 g; MgSO_4_ · 7H_2_O 0.02 g; NH_4_NO_3_ 1 g; KH_2_PO_4_ 1 g; K_2_HPO_4_ 1 g; FeCl_3_ ∙ 6H_2_O 0.005 g; H_2_O 1 l, with pH 7) containing 5 mg l^−1^ of BTEX in total (conc. of each BTEX was ~ 0.83 mg l^−1^) was inoculated with 70 μl of *P. veronii* BFHA4_7 bacterial suspension (OD_600nm_ = 1) obtained in saline solution (0.9% NaCl). According to our preliminary studies, this inoculum volume corresponds to ~ 5·10^6^ *P. veronii* BFHA4_7 cells per ml test solution (data not shown). The obtained microcosms were incubated at 28 °C while shaking at 145 rpm. The goal at this point was to assess whether *P. veronii* strain BFHA4_7 was capable of degrading all the six BTEX compounds cometabolically (cometabolism—simultaneous degradation of two compounds, in which the degradation of the secondary substrate depends on the presence of the primary substrate; Joshua et al. 2011). The experiments were conducted in triplicates in the presence of abiotic control samples.

#### Testing BTEX biodegradation ability of the co-cultured strains

As mentioned earlier, strain BFB1_13 was capable of degrading all six BTEX after only 168 h of incubation (Benedek et al. 2021). In addition, out of the six BTEX compounds, strain BFHA4_7 degraded only toluene, *m*- and *p*-xylene, cometabolically as well, as this will become apparent in later sections of this study. The goal here was to test whether the two organisms are more efficient together in BTEX biodegradation or not. It was highly expected that there will be catabolic cooperation and the two bacteria complement each other in the BTEX degradation ability leading to faster and more efficient biodegradation of the full range of BTEX.

The tests were conducted in triplicates in 1 L hermetically closed crimp sealed serum bottles containing 700 ml of the aforementioned mineral salts solution supplemented initially with a mixture of BTEX compounds of 10 mg l^−1^ in total (conc. ~ 1.7 mg l^−1^ for each BTEX). Test solutions were co-inoculated with 1–1 ml of each BFB1_13 and BFHA4_7 bacterial suspensions of OD_600nm_ = 1 (~ 10^6^–10^6^ bacterium cells per ml test solution). In the case of complete biodegradation, BTEX supplementations took place. Starting from the second supplementation, 20 mg l^−1^ of BTEX was added to the microcosms in total (conc. ~ 3.3 mg l^−1^ for each BTEX). The co-culture microcosm experiment lasted for 8 days with six supplementations in total. Right before the 3rd and 6th BTEX supplementation 60 ml of sterile air was introduced into the bottles to maintain aerobic conditions. 24 h after the 6th supplementation the 3rd aeration with injection of 120 ml of sterile air took place.

At the end of the microcosm experiment, the relative abundance of the co-cultured strains was determined by CFU determinations and (ii) 16*S rRNA* gene-based T-RFLP analysis (for more details please see section Co-cultivability of the studied strains in BTEX-amended mineral salts solution).

#### Testing the biodegradation capacity of the consortium on a real BTEX-contaminated environmental sample

At this stage of the study, using the two-strain consortium the efficiency of allochthonous bioaugmentation was tested on BTEX biodegradation in a real contaminated groundwater containing the autochthonous microbial community. The microcosm experiments were conducted in triplicates in 1 L hermetically closed crimp sealed serum bottles containing 700 ml of BTEX-contaminated groundwater originating from the “Siklós”-contaminated site of Hungary, well ST/2, sampled on 23rd of March 2021 (the “Siklós”-site was thoroughly described by Táncsics et al. 2012). Groundwater samples were collected according to MSZ ISO 5667-11:2009/1-2 standard and stored in a cooling box and later on at 4 °C. Samples were processed 24 h after arrival to the laboratory. Original qualitative and quantitative parameters of the contaminated groundwater regarding general water chemistry and hydrocarbon pollutants were determined by an accredited laboratory, the Wessling Hungary Ltd. To determine specific electrical conductivity and groundwater pH MSZ EN ISO 27888:1998 and MSZ ISO 10523:2003 standards were used, respectively. For determination of sulphate and nitrate concentrations MSZ EN ISO 10304-1:2009, for Fe^2+^ and Mn^2+^ amounts EPA Method 200.8:1999 and MSZ EN ISO 17294-2:2005 analytical standards were used. To determine the concentration of TPHs (C5-C40) and BTEX, WBSE-26:2009 and MSZ 1484-7: 2009 analytical standards and HP-6890-GC FID/PID (Agilent, USA) apparatus were used. Redox potential and temperature values were determined on site by using HI 9829 multiparameter meter (Hanna Instruments). The dissolved oxygen concentration was also determined on site using a HQ30D portable meter (Hach).

At the beginning of the microcosm experiment, to the already contaminated groundwater samples containing the pristine autochthonous microbial community, 10 mg l^−1^ of extra BTEX mixture was added (spiked) in total (conc. of each BTEX compound was ~ 1.7 mg l^−1^). Microcosms were co-inoculated with 10–10 ml of each *V. paradoxus* BFB1_13 and *P. veronii* BFHA4_7 bacterial suspensions of OD_600nm_ = 0.9. Inoculants were obtained in mineral salts solution by using 48 h old cultures developed on R2A plates, revitalized from -80 °C stocks. The inoculated groundwater samples contained per ml ~ 10^7^–10^7^ BFB1_13 and BFHA4_7 bacterium cells.

Non-inoculated microcosms containing the intact autochthonous microbial community were initiated as well under the same circumstances.

To test if the BTEX biodegradation capacity of the revitalized cells still exists, mineral salts solutions (50 ml) containing 10 mg l^−1^ BTEX were co-inoculated with 100–100 μl of BFB1_13 and BFHA4_7 bacterial suspensions obtained in the previous step (OD_600nm_ = 0.9).

To mimic close to in situ conditions, all the microcosms were incubated at 15 °C under static conditions in the absence of light.

The concentration of BTEX throughout the microcosm experiments was determined from the headspace of the bottles by using an SPME polydimethylsiloxane fiber assembly (Supelco) for sampling. Before GC–MS measurements, 1 h was left for the BTEX compounds added to the microcosms to reach the equilibrium between the liquid and gas phases (steady-state). Trace 1300 gas chromatograph coupled to ISQ Single Quadrupole mass spectrometer (ThermoFisher Scientific, US) was used for analysis. During the measurement, injector and detector temperatures were maintained at 200 °C and 250 °C, respectively. The oven temperature program was set to 40 °C for 3 min, then ramped at a rate of 20 °C min^−1^ to 190 °C and finally held for 1 min. Helium was used as carrier gas at a flow rate of 1.2 ml min^−1^. SLB™-5 ms fused silica capillary column was used for separation (30 m × 0.25 mm × 0.25 μm, Sigma-Aldrich, Supelco). The mass spectrometer was operated at full scan mode.

### Testing co-cultivability of the studied bacterial isolates

Previously, the optimal cultivation conditions for the strains were determined (data not published). According to those results, for the cultivation of both strains modified nutrient-broth (yeast extract 2 g, meat extract 1 g, casein peptone 5 g, NaCl 10 g, pH 7–8, H_2_O 1 L) incubated at 28–30 °C is the most appropriate. Optimization of cultivation conditions showed that compared to *P. veronii* BFHA4_7 strain *V. paradoxus* BFB1_13 grows slower in nutrient-broth (Additional file 1: Fig. S3). During co-cultivability tests in nutrient rich medium the difference between the growth rates was taken into consideration.

#### Determination of strain specific 16S rRNA gene-based T-RFLP profiles

Prior to the co-cultivation experiments, the optimal parameters for the terminal restriction fragment length polymorphism analyses (T-RFLP) were also assessed. T-RFLP is a highly reproducible, quantitative and qualitative molecular method for rapid analysis of microbial communities. Using the *16S rRNA* gene-based T-RFLP technique, changes in microbial community structure that occur on temporal or spatial scales can be assessed (Liu et al. 1997). In this study, T-RFLP was used to determine the co-cultivability and temporal dynamics of the studied isolates during the co-cultivation experiments.

To obtain the T-RFLP profiles of the studied strains as a first step, genomic DNA of pure isolates (24–48 h old cultures) was extracted by using DNeasy UltraClean Microbial Kit (Qiagen) according to the instructions of the manufacturer. As a second step, *16S rRNA* genes were PCR amplified by using Bacteria specific fluorescently labelled 27-VIC-F (5′-AGA GTT TGA TCC TGG CTC AG-3′) forward and non-labelled 1492-R (5′-TAC GGY TAC CTT GTT ACG ACT T-3′) reverse primers. The conditions of PCR and the used temperature profile were the same as described by Táncsics et al. (2012). Subsequently, 2 μl of the obtained fluorescently-labelled *16S rRNA* gene amplicons were digested with 3 U of FastDigest RsaI (5′-GT/AC-3′) and 1 U of AluI (5′-AG/CT-3′) restriction endonuclease at 37 °C for 15 min and 1.5 h, respectively (ThermoFisher Scientific). The generated fluorescently-labelled terminal restriction fragments (T-RFs) were purified by ethanol precipitation, separated and detected by capillary gel electrophoresis using an ABI 3130 Genetic Analyzer (Applied Biosystems). The applied internal lane size standards were GeneScan™ 500 LIZ™ and GeneScan™ 1200 LIZ™ (Applied Biosystems). T-RFLP electropherograms were analyzed with the GeneMapper Software v4.0 (Applied Biosystems).

#### Co-cultivation of the studied strains in nutrient rich growth medium

For the co-cultivation of isolates nutrient-broth (pH = 7 and 1% NaCl) was used and incubated at 30 °C and shaken at 160 rpm. As a first step, actively growing cultures of *P. veronii* BFHA4_7 and *V. paradoxus* BFB1_13 were obtained separately on nutrient-agar for 24 and 48 h, respectively. By using physiological saline solution (0.9% NaCl) bacterial suspensions of OD_600nm_ = 0.5 were obtained, containing 2.3 ‧ 10^8^ and 2.97 ‧ 10^8^ cells per ml saline solution for strains BFB1_13 and BFHA4_7, respectively (data obtained earlier, not shown). One liter of nutrient-broth was inoculated first with 100 μl of BFB1_13 bacterial suspension, 72 h later 100 μl of BFHA4_7 bacterial suspension was added to the culture. At this step, the faster growth rate of *P. veronii* strain BFHA4_7 was taken into account, as we wanted to avoid over proliferation of strain BFHA4_7 at the expense of strain BFB1_13. After 1 week of incubation, 10 ml of the bacterial suspension was transferred to freshly prepared and sterilized nutrient-broth (1 L) and incubated for one more week. From the one- and two-week-old bacterial cultures, 15–15 ml were centrifuged at 2360*g*. The community DNA, originating from the two strains, was extracted from the pellet by using DNeasy UltraClean Microbial Kit (Qiagen) and community T-RFLP analysis was conducted as described above by using RsaI endonuclease. Community T-RFLP profiles for each week were compared and temporal dynamics were assessed.

#### Co-cultivability of the studied strains in BTEX-amended mineral salts solution

After 8 days of testing the biodegradation capacity of the co-cultured strains, and after a series of BTEX concentration reductions and supplementations, the relative abundance of the co-cultured strains at the end of the experiment was determined by (i) the enumeration of viable bacterial cells (CFU determinations) and (ii) by *16S rRNA* gene-based T-RFLP as described above.

The determination of bacterial cell numbers specific to *P. veronii* BFHA4_7 and *V. paradoxus* BFB1_13 was simple since the two strains showed distinct colony morphologies (Additional file 1: Fig. S2). After 8 days of incubation, the obtained bacterial suspensions from each replicate were serially diluted up to 10^8^. Subsequently, 100 μl from each dilution was inoculated onto a nutrient rich medium. The developed strain specific colonies were enumerated after 72 h of incubation and represented on bar charts.

Before T-RFLP analysis, 50–50 ml of co-cultures were centrifuged at 2360*g* and the community DNA from the two strains was extracted from the pellet by using DNeasy UltraClean Microbial Kit (Qiagen). Fluorescently-labelled T-RFs originating from the two-strain community were generated by using AluI endonuclease and analyzed as described above*.*

## Results

### Results of BTEX biodegradation studies

#### BTEX mixture biodegradation capacity of *P. veronii* BFHA4_7

Similar to the results of individual BTEX biodegradation (Benedek et al. 2018), in the presence of all six BTEX compounds, strain BFHA4_7 was capable of degrading only toluene, *m*- and *p*-xylene. Complete biodegradation occurred in a relatively short period of time, after 96 h of incubation (Additional file 1: Fig. S1). In the case of the remaining BTEX, no noticeable biodegradation occurred by the end of the experiment. *P. veronii* strain BFHA4_7 could not degrade benzene, ethylbenzene and *o*-xylene cometabolically either.

#### BTEX biodegradation capacity of the two-strain consortium in mineral salts solution

**Fig. 1:**
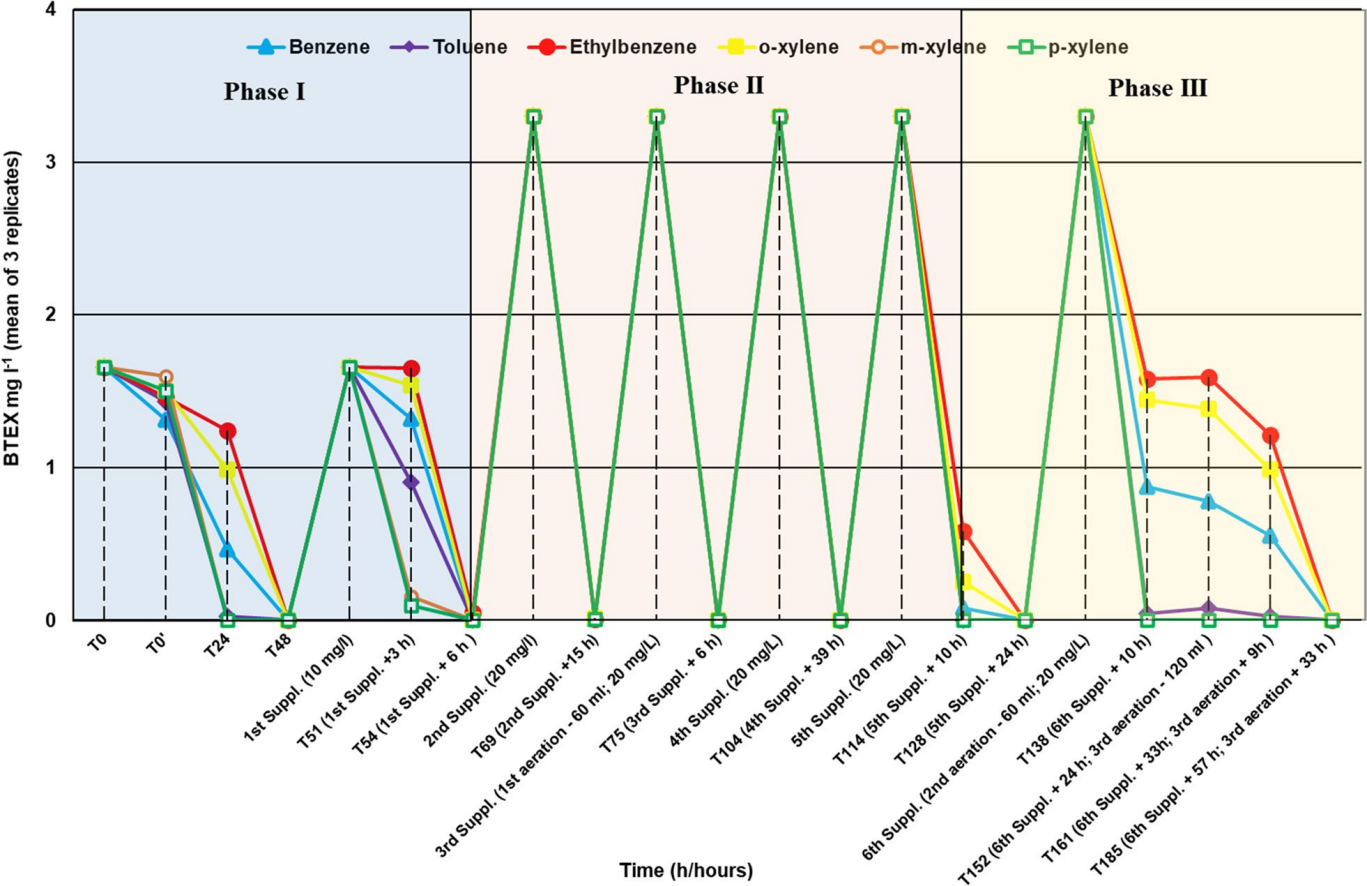
BTEX biodegradation capacity of the co-cultured strains, *P. veronii* BFHA4_7 and *V. paradoxus* BFB1_13, as assessed in mineral salts solution. The mean of three replicates is shown without indicating on the figure standard deviation values (STD) for a better clarity. Precise BTEX concentrations together with STD values determined at each timepoint are shown in Additional file 1: Table S1. Phase I Total concentration of BTEX used for supplementations was 10 mg l^−1^. Phase II Total concentration of BTEX used for supplementations was 20 mg l^−1^ Phase III Slightly reduced BTEX biodegradation capacity of the two-strain community. T0—Concentration of BTEX shortly after their addition to the test solutions. T0’—Concentration of BTEX after reaching steady-state, incubation of bottles for 1 week before bacterial inoculation

Within the inoculated samples, after 24 h of incubation a significant concentration reduction was observed in toluene, *m*- and *p*-xylene, with the concentration of these compounds decreasing to zero. They were followed by benzene, *o*-xylene and ethylbenzene showing a concentration reduction to 0.5 ± 0.1, 1 ± 0.1 and 1.2 ± 0.1 mg l^−1^, respectively. After 48 h, all the BTEX were eliminated from the test solutions (total initial conc. 10 mg l^−1^). After the 1st BTEX supplementation, 3 h were left to reach the equilibrium between the liquid and gas phase (steady-state). After 3 h, notable *p*- and *m*-xylene concentration reductions occurred (conc. red. to 0.1 ± 0.1 and 0.2 ± 0.1 mg l^−1^, respectively), followed by toluene (conc. red. to 0.9 ± 0.2 mg l^−1^) and *o*-xylene (conc. red. to 1.5 ± 0.2 mg l^−1^). Three hours later, the concentration of all BTEX reduced to zero (in total 6 h after the 1st supplementation; end of Phase I). Fifteen hours after the 2nd supplementation (total initial conc. of BTEX was 20 mg l^−1^) the concentration reduction of BTEX compounds was at least 99.4%. Six hours after the 3rd supplementation and I. aeration the concentration of all BTEX reduced to zero. Up until the 104th hour, the degradation of BTEX (20 mg l^−1^) was complete. However, 10 h after the 5th supplementation the microcosms showed a slightly reduced BTEX degradation capacity (end of Phase II), but still fourteen hours later the concentration of BTEX reduced to zero again (Fig. 1; Additional file 1: Table S1).

Ten hours after the 6th supplementation and 2nd aeration, complete or near complete elimination was observed in the case of *m*- and *p*-xylene, as well as toluene. Meanwhile, the concentration of benzene, *o*-xylene and ethylbenzene reduced to 0.9 ± 0.1, 1.4 ± 0.1 mg l^−1^ and 1.6 ± 0.2 mg l^−1^, respectively. 23 h later, no remarkable change in benzene, *o*-xylene and ethylbenzene reductions were recorded, notwithstanding a 3rd aeration with 120 ml of sterile air injection was conducted. After 57 h of the last BTEX supplementation and 33 h of the 3rd aeration the concentration of BTEX reduced to zero again (end of Phase III; Fig. 1; Additional file 1: Table S1).

#### Testing the biodegradation capacity of the consortium on a real BTEX-contaminated environmental sample (groundwater)

As determined prior to the microcosm experiments, the groundwater sample initially contained 0.1, 0.8, and 3.2 mg l^−1^ of benzene, ethylbenzene and xylenes, respectively. Initially, the concentration of toluene was very low 0.009 mg l^−1^. Overall, the concentration of total alkyl benzenes was high 7.2 mg l^−1^.

In the contaminated groundwater, reducing conditions prevailed as indicated by the low dissolved oxygen and redox potential values and high Fe(II) concentration. The temperature of the groundwater was 13 °C with a close to neutral pH (Table 1).

**Table 1:** Results of groundwater chemical analyses at the date of sampling—general water chemistry and the concentration of main petroleum hydrocarbons are shown

General groundwater chemistry and pollutants	Unit	Contaminated well ST/2
Specific electrical conductivity	μS cm^−1^	1360
Redox potential	mV	− 19
Dissolved oxygen	mg l^−1^	0.8
pH	–	6.5
Temperature	°C	13
SO_4_^2−^	mg l^−1^	< 30
NO_3_^¯^	mg l^−1^	< 5
Fe(II)	mg l^−1^	9.4
Mn(II)	mg l^−1^	4.6
TPHs^a^	mg l^−1^	1.9
Benzene	mg l^−1^	0.1
Toluene	mg l^−1^	0.009
Ethylbenzene	mg l^−1^	0.8
Xylenes	mg l^−1^	3.2
Other alkyl benzenes	mg l^−1^	7.2

^a^TPH—Total Aliphatic Petroleum Hydrocarbons (C_5_–C_40_)

**Fig. 2:**
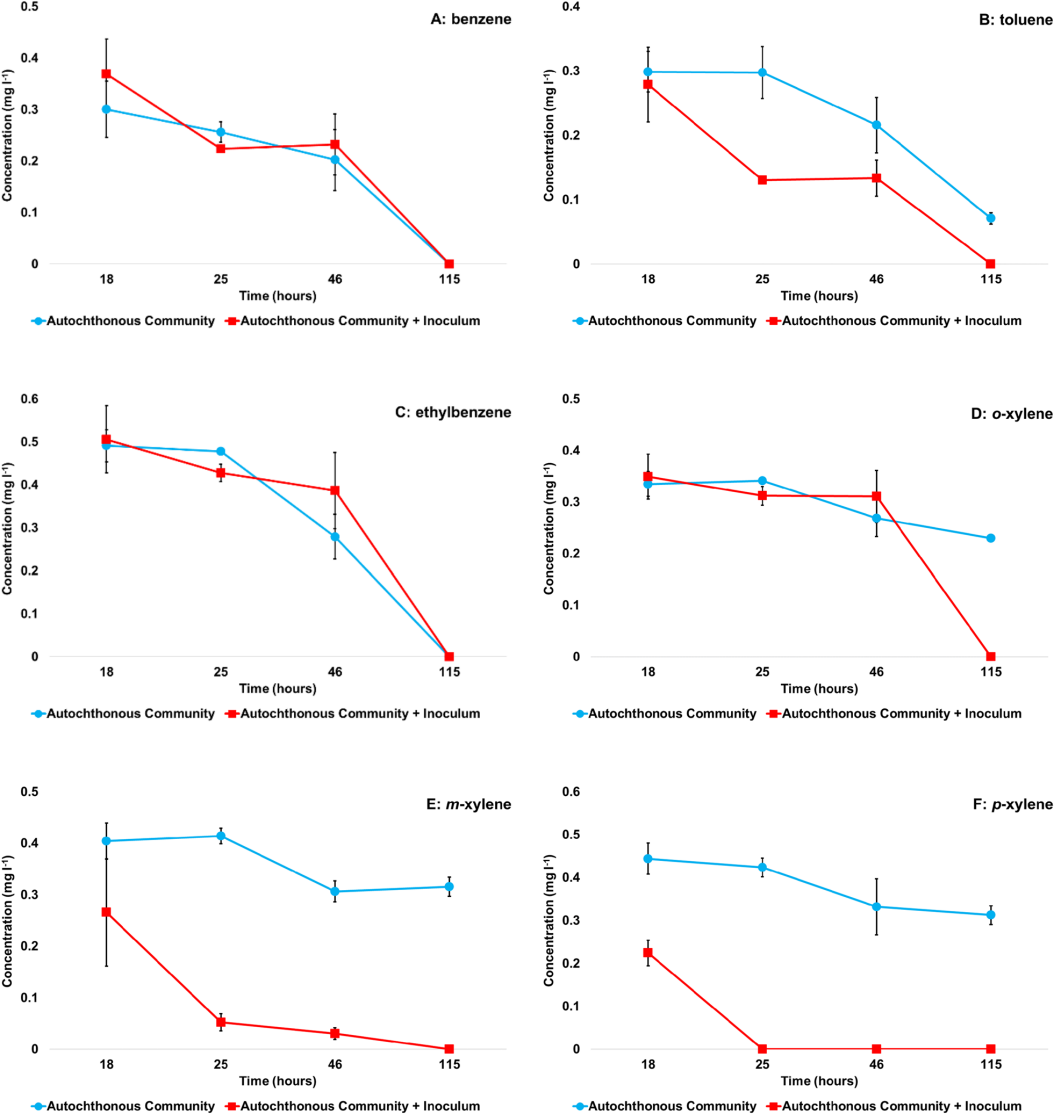
BTEX biodegradation capacity of microcosms containing solely the autochthonous bacterial community (blue lines, circle) and the autochthonous bacterial community inoculated with *V. paradoxus* BFB1_13 and *P. veronii* BFHA4_7 strains (Inoculum; red lines, square). The time frame between the 18th and 115th hours of incubation is shown. At each time point mean BTEX concentrations together with standard deviation values represented as error bars are depicted. When error bars are not visible, they are hidden behind the symbols (Additional file 1: Table S2). **A** Benzene; **B** Toluene; **C** Ethylbenzene; **D** *o*-xylene; **E** *m*-xylene; **F** *p*-xylene

The microcosm experiments spiked with 10 mg l^−1^ of BTEX were run for 5 days (115 h) in static cultures incubated at 15 °C in the dark. As shown in Fig. 2A and C, no remarkable difference was observed in the biodegradation of benzene and ethylbenzene between microcosms containing either the autochthonous bacterial community or the autochthonous bacterial community inoculated with *V. paradoxus* BFB1_13 and *P. veronii* BFHA4_7. The rate of biodegradation of these compounds was similar in both settings, after 115 h of incubation the concentration of benzene and ethylbenzene decreased to zero. In the case of other BTEX compounds, as compared to the autochthonous community, the inoculated microcosms showed a faster biodegradation rate. Throughout the whole experiment, in the case of toluene, *m*- and *p*-xylene, the inoculated groundwater samples (Autochthonous Community + Inoculum) showed faster biodegradation rates compared to non-inoculated samples. The positive effect of inoculation on *o*-xylene biodegradation manifested only after 115 h of incubation. In the inoculated samples, the concentration of *o*-xylene decreased to zero, whereas in the microcosms containing only the autochthonous bacterial community the remaining *o*-xylene concentration was 0.2 ± 0.01 mg l^−1^ (Fig. 2D). During the whole experiment, the most striking difference between inoculated and non-inoculated groundwater microcosms was observed in the case of *m*- and *p*-xylene, especially *p*-xylene biodegradation (Fig. 2E and F). After 115 h of incubation, in the inoculated samples the concentration of *m*-xylene decreased to zero. However, a remarkable concentration reduction was recorded already after 25 h of incubation (Fig. 2E). At the end of the experiment, in the non-inoculated samples the concentration of *m*-xylene was still 0.3 ± 0.02 mg l^−1^ (Additional file 1: Table S2).

The fastest biodegradation rate was observed in the case of *p*-xylene. In the inoculated groundwater samples, the concentration of *p*-xylene decreased to zero already after 25 h of incubation. In the meantime, at the end of the experiment in the non-inoculated microcosms containing only the native microbial community the concentration of *p*-xylene was still 0.3 ± 0.02 μg l^−1^ (Fig. 2F).

### Results of co-cultivability studies

#### *T-RFLP profiles of isolates obtained with *RsaI* and *AluI* restriction enzymes*

*16S rRNA* gene-based T-RFLP profiles of *V. paradoxus* strain BFB1_13 and *P. veronii* strain BFHA4_7 pure cultures were determined. According to the results, using RsaI restriction endonuclease, on the T-RFLP electropherograms a ~ 420 bp sized T-RF designates strain BFB1_13 and a T-RF of ~ 800 bp indicates strain BFHA4_7 (Additional file 1: Fig. S4 A and B). By using AluI enzyme T-RFs of ~ 70 bp and ~ 148 bp designate strains BFHA4_7 and BFB1_13, respectively (Additional file 1: Fig. S4C and D).

#### Co-cultivability of the isolates in nutrient rich medium

Unexpectedly, the T-RFLP analysis indicated that in a nutrient rich medium, strain BFHA4_7 overproliferated strain BFB1_13 even though it was inoculated into the medium 72 h later. After 1 week of incubation, the relative abundance of strain BFHA4_7 affiliated T-RF (80.7 ± 3.6%) was four times higher than that of strain BFB1_13 (19.3 ± 3.6%). After 2 weeks of co-cultivation the strain BFB1_13 related T-RF almost completely disappeared from the nutrient rich co-cultures (Additional file 1: Fig. S5).

#### Results of co-cultivability of strains in BTEX-amended mineral salts solution

**Fig. 3:**
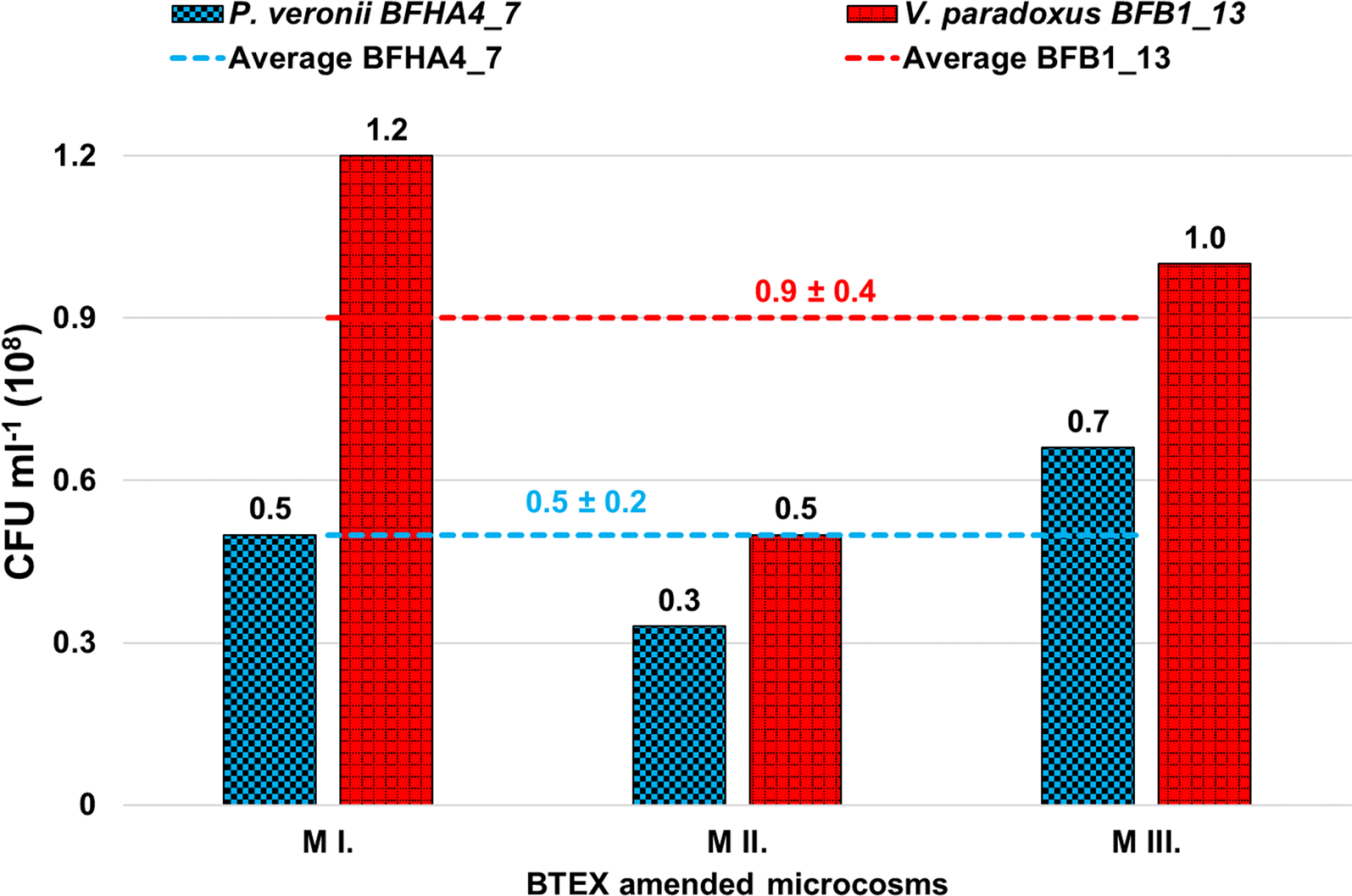
Results of colony forming unit (CFU) determination of *P. veronii* BFHA4_7 and *V. paradoxus* BFB1_13 strains re-isolated from the BTEX amended (total conc. 10–20 mg l^−1^) mineral salts solution microcosms (M; I. II. and III. replicates). Re-isolations took place after 8 days of incubation

Compared to co-cultivability tests conducted in nutrient rich medium, quite different results were obtained regarding the co-cultivability of strains in mineral salts solution in the presence of BTEX as a sole source of carbon and energy. After 8 days of incubation in mineral salts solution amended with high concentrations of BTEX, and after a series of BTEX depletions and supplementations, both strains could be re-isolated from the test media in equally high numbers. According to CFU determinations, on average 9 ± 3.6 ‧ 10^7^ and 5.5 ± 1.7 ‧ 10^7^ V*. paradoxus* BFB1_13 and *P. veronii* BFHA4_7 bacterial cells were present per ml co-cultures, respectively (Fig. 3).

In contrast to the results obtained in nutrient rich medium, at the end of the experiment the CFU per ml of *V. paradoxus* strain BFB1_13 was higher than that of *P. veronii* strain BFHA4_7; on average a 1.63-fold difference in CFU was found.

The results of T-RFLP analysis supported the results of CFU determinations. At the end of the experiment, both strains could be detected on the community-based T-RFLP electropherograms. The relative abundance of *V. paradoxus* BFB1_13 and *P. veronii* BFHA4_7 affiliated T-RFs were 62.6 ± 8.3% and 37.5 ± 8.3%, respectively. Similar to CFU determinations, a 1.67-fold difference in the relative abundance between the strains was recorded in favor of strain BFB1_13 (Additional file 1: Fig. S6).

## Discussion

Bioremediation is still the most advantageous approach for the elimination of BTEX pollutions. Within bioremediation, bioaugmentation has an outstanding place, and it has been defined as the inoculation of microorganisms with the capability to degrade the target pollutants in a contaminated environment (Maier 2000; Heinaru et al. 2005). The inoculation can be conducted with one single strain or with a consortium of microbial strains with diverse metabolic capacities. Bioaugmentation has been proposed to be applied in those cases where biostimulation and natural attenuation are ineffective (Iwamoto and Nasu 2001; El Fantroussi and Agathos 2005). Inoculation with microorganisms harboring the necessary metabolic pathways for the degradation of the target pollutants can accelerate the removal rate and, hence, reduce the time and capital required for the remediation (Nowak and Mrozik 2016; Ławniczak et al. 2020). However, the success of microbial cell bioaugmentation highly depends on the survival rate and maintenance of catabolic activity of the microbial strains introduced into the contaminated environment (Singh and Ward 2004). The inoculated strains must compete with the autochthonous microbial populations already present at the contaminated sites regarding the usage of sources of energy, nutrients and electron acceptors (Ławniczak et al. 2020). Furthermore, the inoculated strains must stand the harsh abiotic conditions dominating the contaminated environment too. Both biotic and abiotic factors may lead to high mortality of the inoculated microorganisms, introduced bacteria may become undetectable soon after inoculation (Garbisu et al. 2017).

In this study, a novel bacterial consortium made up of strains *V. paradoxus* BFB1_13 and *P. veronii* BFHA4_7 was developed for rapid and complete biodegradation of BTEX compounds. It must be emphasized that although bacterial consortia for BTEX removal do exist in general, these inoculants were tested mostly either under laboratory conditions (room temperature or above, shaking cultures, absence of autochthonous community) or without studying biodegradation of all three xylene isomers separately (Attaway and Schmidt 2002; Shim et al. 2002; Deeb and Alvarez-Cohen 1999; Arafa 2003; Farhadian et al. 2008; Mazzeo et al. 2010; Fellie et al. 2012; El-Naas et al. 2014; Nagarajan and Loh 2015). For the sake of completeness, it should be pointed out that the applied total BTEX concentration in this study (10–20 mg l^−1^) was below the applied concentrations in previous studies mentioned above (50–600 mg l^−1^), however from an environmental pollution point of view it was still in the range of high BTEX contamination. Bacterial consortia containing species such as *Variovorax* sp. and *Pseudomonas* sp. do exist and are used for the degradation of e.g., linuron and 2,4-dinitrotoluene (Dejonghe et al. 2003; Snellinx et al. 2003). According to Satola et al. (2013) *V. paradoxus* affiliated isolates are capable of degrading a wide variety of recalcitrant organic pollutants such as PAHs, PCBs, dinitrotoluene, and trichloroethylene. Until recently (Benedek et al. 2021), there was no information regarding the BTEX biodegradation capacity of *V. paradoxus* pure cultures, although members of the genus *Variovorax* are often detected at petroleum hydrocarbon contaminated sites where the primary contaminants are simple aromatic hydrocarbons such as BTEX (Hendrickx et al. 2006; Benedek et al. 2016 and 2018; Posman et al. 2017). On the other hand, based on studies dealing with the metabolism and genetics of bacteria degrading simple aromatic hydrocarbons, it is evident that *Pseudomonas* species are highly involved in the degradation of BTEX (Arenghi et al. 2001; Hendrickx et al. 2005; Wang et al. 2008). Moreover, based on the literature, isolates affiliating with *P. veronii,* besides BTEX compounds*,* are able to use as a sole source of carbon and energy different hydrocarbons, including phenolic compounds, alkyl methyl ketones, chlorinated aromatic hydrocarbons and dioxins (Ajithkumar et al 2003; Nam et al. 2003; Hong et al. 2004; Onaca et al. 2007; Morales et al. 2016).

Based on the above mentioned, it is reasonable to develop such a bacterial consortium that according to the obtained results is very effective in BTEX biodegradation under laboratory conditions and close to in-situ conditions as well in the presence of the autochthonous microbial community of BTEX-contaminated groundwater. Although in nutrient rich medium *P. veronii* strain BFHA4_7 overproliferated *V. paradoxus* strain BFB1_13, in BTEX amended mineral salts solution the proportions in relative abundance changed in favor of strain BFB1_13. From Fig. 1. it is evident that the biodegradation of BTEX by the consortium is initiated by *P. veronii* BFHA4_7. First BTEX compounds whose concentration decreased to zero were *p*-, *m*-xylene and toluene, mainly specific to strain BFHA4_7 (Phase I and III). The degradation of BTEX compounds specific to strain BFB1_13 (benzene, ethylbenzene and *o*-xylene) also occurred but was slightly delayed. The synergistic degradation of BTEX by the obtained consortium is supported on the one hand by these findings and also by the fact that all the six BTEX compounds were completely degraded from the bottles and at the end of the microcosm experiment both strains could be re-isolated in high number (10^8^ CFU ml^−1^). In the co-inoculated mineral salts solution, BTEX biodegradation occurred in as little as 6 h. However, it has to be mentioned that rapid BTEX biodegradation was observable until the 104th hours of incubation after which a reduction in BTEX biodegradation capacity of the consortium occurred (Fig. 1). Decreased biodegradation ability of the strains can be explained with the intervening of a weekend when no BTEX supplementation took place for 40 h, from Saturday afternoon until Monday morning. Presumably, the BTEX supplemented on Saturday afternoon were quickly degraded and for the rest of the weekend the culture left without carbon and energy source, and entered the stationary phase. However, according to Phase III (Fig. 1) the BTEX biodegradation capacity of the two-strain community revived and 57 h after the last BTEX supplementation the concentration of BTEX reduced again to zero. Accordingly, the consortium has the ability to quickly reactivate its BTEX biodegradation capacity after a carbon source starvation period. All the above-mentioned results and the fact that *V. paradoxus* BFB1_13 did not disappear from the BTEX supplemented test solutions and exceeded in cell number *P. veronii* strain BFHA4_7 by the end of the experiment, may indicate that the two strains complement their metabolic capacities in BTEX biodegradation and work in synergy to gain energy from the breakdown of BTEX compounds specific to them.

Most importantly, under close to in situ conditions the consortium proved to be effective in BTEX biodegradation in a real BTEX-contaminated groundwater too, containing the autochthonous microbial community (total conc. of applied BTEX was ~ 10 mg l^−1^). The allochthonous bioaugmentation with the engineered consortium proved successful. The “Siklós”-contaminated site existed for more than twenty years and the autochthonous community had enough time to adapt to the contaminants, according to the results of analytical chemistry the concentration of alkyl-benzenes, including BTEX except for toluene, is still high in the groundwater (Table 1). Based on Fig. 2, the inoculation with the two-strain consortium positively impacted the BTEX biodegradation process, taking place in the groundwater samples. Regarding benzene, toluene and ethylbenzene no remarkable beneficial effect of the inoculation was found compared to the activity of the autochthonous community. In the case of xylenes’ biodegradation, the positive effect of the inoculation was pronounced. These results indicate that most probably the autochthonous microbial community in the groundwater inherently harbors benzene, toluene and ethylbenzene biodegradation capacity, but the xylenes elimination potential of the community is low. In the inoculated groundwater samples complete or near complete *p*- and *m*-xylenes biodegradation occurred within 25 h of incubation (BTEX compounds specific to *P. veronii* BFHA4_7), whereas in the microcosms containing solely the autochthonous community, the concentration of these compounds was still 0.4 mg l^−1^ (400 μg l^−1^). After 115 h of incubation, complete *o*-xylene biodegradation occurred (specific to *V. paradoxus* BFB1_13) only in the case of inoculated groundwater microcosms. At this point, *o*-xylene concentration in the non-inoculated groundwater microcosms was 0.23 mg l^−1^ (230 μg l^−1^). Even after 2 weeks of incubation, in the non-inoculated groundwater samples the concentration of *o*-, *m*-, and *p*-xylene was 128.9, 73.7 and 52.2 μg l^−1^, respectively. Based on the aforementioned results, it can be assumed that the biodegradation of xylenes by the autochthonous bacterial community found in the groundwater occurs cometabolically. As long as there are other BTEX compounds in the environment the biodegradation of xylenes is efficient. As soon as benzene, toluene and ethylbenzene are being depleted, the biodegradation of xylenes ceases. Cometabolic xylene biodegradation has been demonstrated earlier. According to Littlejohns and Daugulis (2008) *o*-xylene was the most resistant against biodegradation by a bacterial consortium containing seven *Pseudomonas* species. The obtained results indicated that *o*-xylene, unlike benzene, toluene and ethylbenzene, was not metabolized by the consortium when being the only carbon source present. It was cometabolized only in the presence of both benzene and toluene. In the presence of ethylbenzene as co-substrate *o*-xylene was not metabolized. In addition, Chang et al. (1993) isolated a *Pseudomonas* sp. strain B1 able to grow on benzene and toluene as the sole sources of carbon and energy. The strain was capable of *p*-xylene biodegradation only cometabolically in the presence of toluene. Results of Miri et al. (2021) also pointed out that *p*-xylene biodegradation by *P. putida* was enhanced in the presence of either toluene or benzene; toluene assimilating cells degraded *p*-xylene more effectively than other substrates.

In summary, it can be concluded that a novel BTEX biodegrading bacterial inoculant was developed applicable under close to in situ conditions, in the presence of the autochthonous microbial community found in a BTEX-contaminated groundwater sample. To the best of our knowledge, such a study, which includes testing the inoculant under close to in situ conditions in the presence of the autochthonous microbial community, for the development of a BTEX-degrading bacterial consortium has not been reported earlier. The obtained two-strain consortium in co-cultures performs better in BTEX biodegradation than in single cultures. Biodegradation of the full range of BTEX occurred faster in co-cultures, and the catabolic cooperation between the two strains was demonstrated. The most significant advantage of this consortium is that, unlike most of other bacterial consortia found in the literature, the role of each member in BTEX biodegradation is clearly defined and the efficiency of the inoculum was also tested on a real BTEX-contaminated groundwater through allochthonous bioaugmentation.

Regarding future perspectives, it would be worth determining the BTEX biodegradation capacity of the obtained bacterial consortium under oxygen-limited conditions (≤ 2 mg l^−1^, O_2_ concentration prevailing at hydrocarbon contaminated sites), again in the presence of the autochthonous microbial community already existing in the contaminated groundwater. It is highly assumed that the consortium will be efficient in BTEX biodegradation also under oxygen-limited conditions since both strains harbor I.2.C *C23Os* involved in oxygen-limited aromatic hydrocarbon biodegradation. Additionally, a long-term microcosm experiment should also be carried out in the presence of the autochthonous community, by conducting a series of BTEX depletions and supplementations, to assess the long-term survival of the inoculant in the presence of the native microbial community.

## Supplementary Information

**Additional file 1.** Supplementary information regarding the colony morphology, T-RFLP profile of the studies isolates, and supplemetary data regarding BTEX-biodegradation capacity of the consortium

## Data Availability

Data generated or analyzed during this study are included in this published article (and its supplementary information files). *V. paradoxus* BFB1_13 and *P. veronii* BFHA4_7 are deposited in the National Collection of Agricultural and Industrial Microorganisms (NCAIM, Budapest, Hungary) under the accession numbers NCAIM B.02666 and NCAIM B. 02670, respectively.
