# Supplementary material for: Development of a bacterial consortium from Variovorax paradoxus and Pseudomonas veronii isolates applicable in the removal of BTEX
Source: AMB Express. 2022 Jan 25;12:4. doi: 10.1186/s13568-022-01349-2 (PMC8787013; doi:10.1186/s13568-022-01349-2)
Supplement: Supplementary file 1 — Additional file 1. Supplementary information regarding the colony morphology, T-RFLP profile of the studies isolates, and supplemetary data regarding BTEX-biodegradation capacity of the consortium [file 13568_2022_1349_MOESM1_ESM.pdf]

Journal Name: AMB Express

Manuscript Title: Development of a bacterial consortium from *Variovorax paradoxus* and *Pseudomonas veronii* isolates applicable in the removal of BTEX

Flóra SZENTGYÖRGYI[a], Tibor BENEDEK[a*], Dzsenifer FEKETE[b], András TÁNCSICS[a], Péter HARKAI[c], Balázs KRISZT[c]

[a]*Hungarian University of Agriculture and Life Sciences, Institute of Aquaculture and Environmental Safety, Department of Molecular Ecology, Gödöllő, H-2100, Páter K. u. 1, Hungary;*

[b]*Soós Ernő Research and Development Center, Circular Economy University Center, University of Pannonia, Nagykanizsa, H-8800, Zrínyi u. 18, Hungary;*

[c]*Hungarian University of Agriculture and Life Sciences, Institute of Aquaculture and Environmental Safety, Department of Environmental Safety, Gödöllő, H-2100, Páter K. u. 1, Hungary;*

*Corresponding author: Tel.: +36-(28)-522-000 ext. 1611

E-mail: benedek.tibor@uni-mate.hu

Number of figures: 6

Number of tables: 2

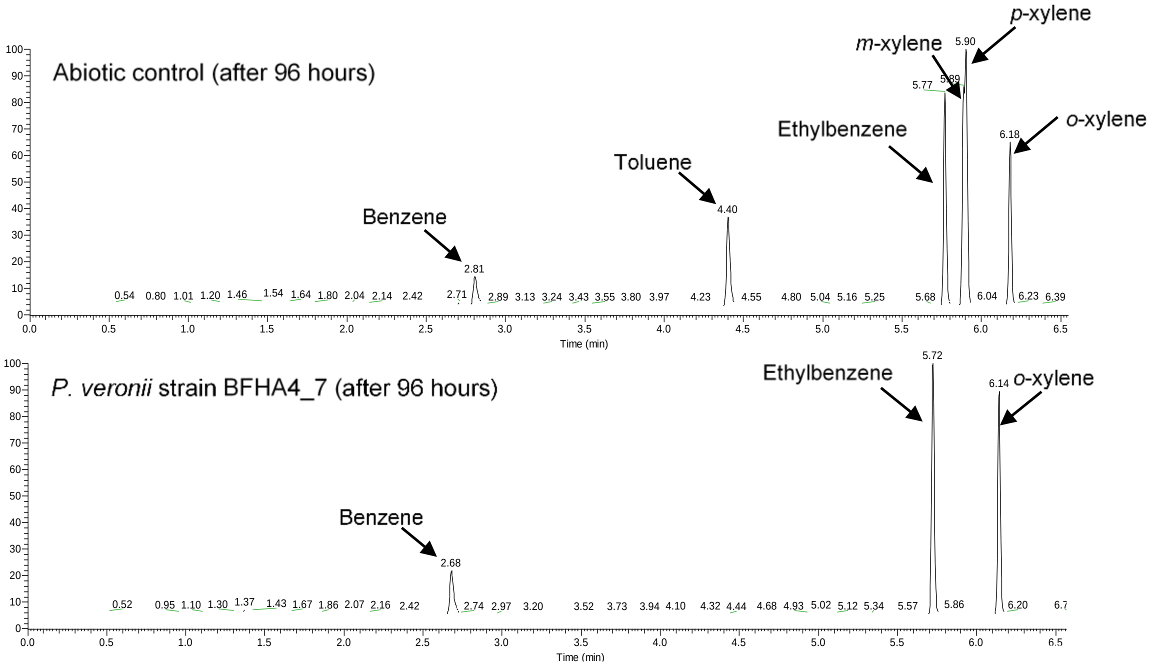

Fig. S1: GC-MS chromatograms showing complete toluene, m- and p-xylene biodegradation by P. veronii strain BFHA4_7 after 96 hours of incubation in mineral salts solution supplemented with BTEX mixture as a single source of carbon and energy

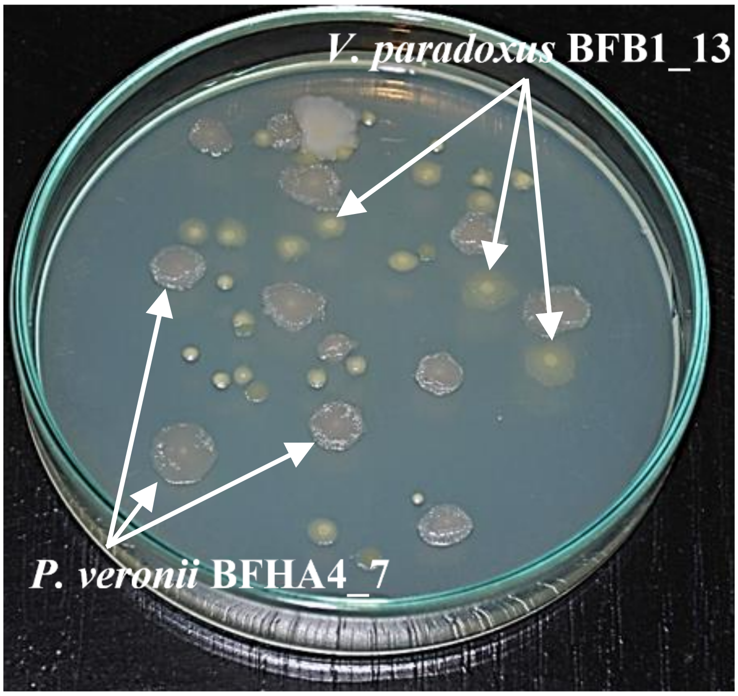

Fig. S2: Colony morphology of the studied bacterial isolates

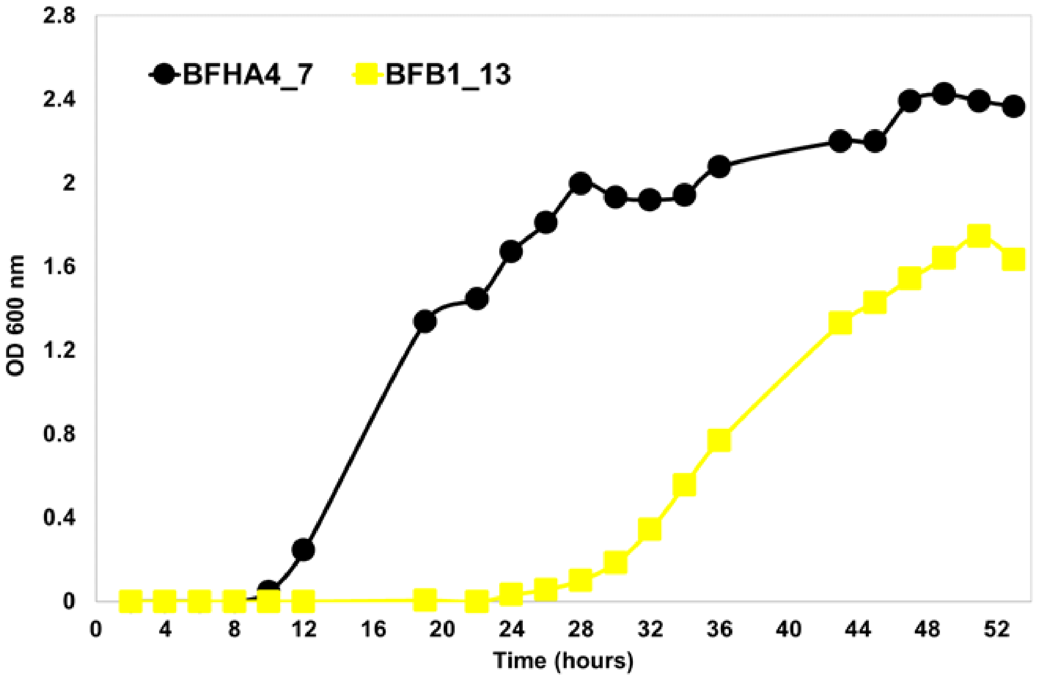

Time (hours)		2	4	6	8	10	12	19	22	24	26	28	30	32	34	36	43	45	47	49	51	53
$OD_{600 nm}$	BFHA4_7	0	0	0	0	0	0.2	1.3	1.4	1.7	1.8	2.0	1.9	1.9	1.9	2.1	2.2	2.2	2.4	2.4	2.4	2.4
	BFB1_13	0	0	0	0	0	0	0	0	0	0.1	0.1	0.2	0.3	0.6	0.8	1.3	1.4	1.5	1.6	1.7	1.6

Fig. S3: Growth curves of *P. veronii* BFHA4_7 (black circle) and *V. paradoxus* BFB1_13 (yellow square) in nutrient-broth incubated at 30 °C, pH 7. OD 600 nm – optical density of the bacterial suspensions measured at 600 nm

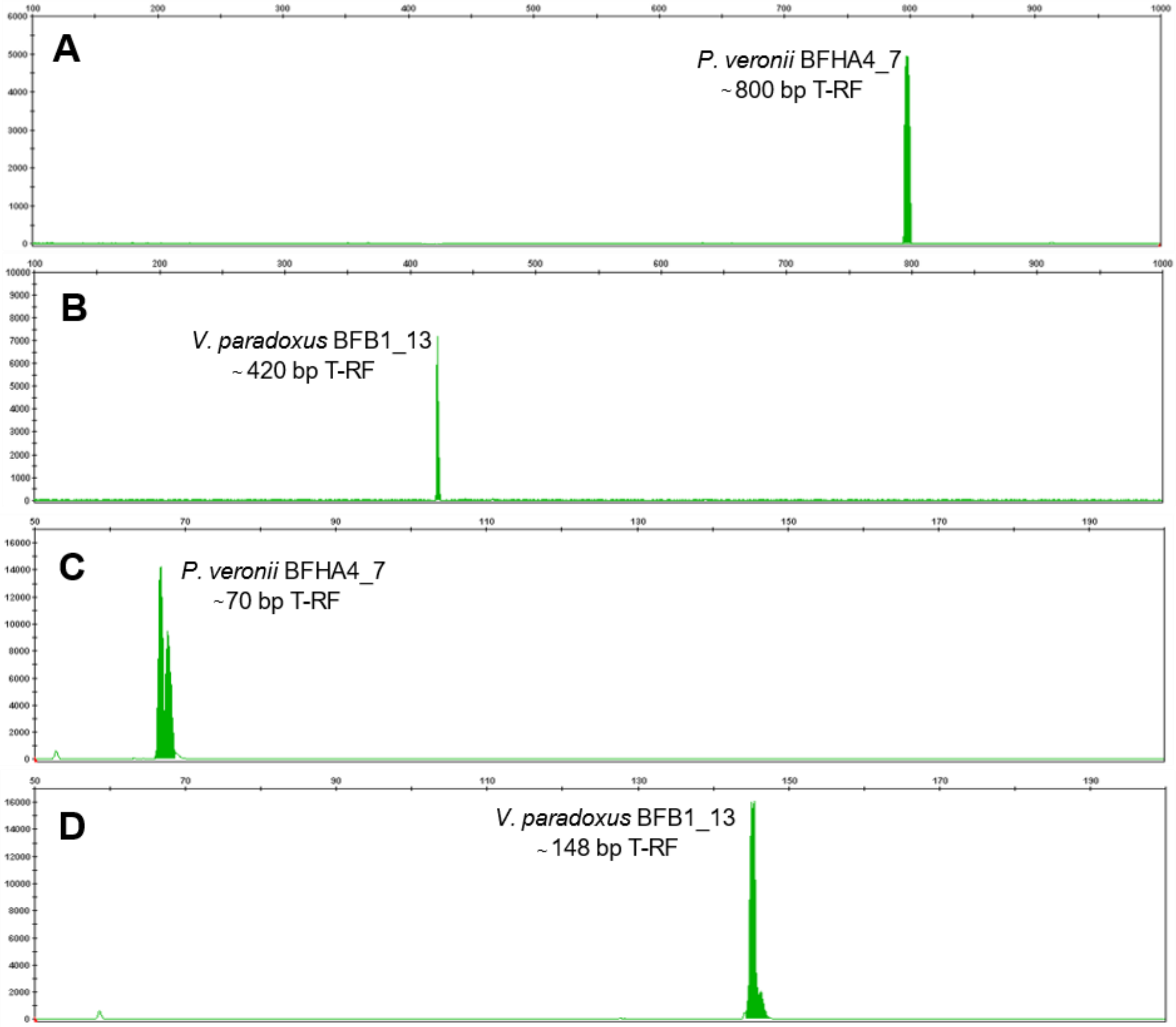

Fig. S4. Representative T-RFLP profiles of pure cultures of *P. veronii* BFHA4_7 and *V. paradoxus* BFB1_13 obtained using *Rsa*I (A and B) and *Alu*I (C and D) restriction endonucleases. x axis – size of T-RFs (bp) according to the internal size standards GeneScan^{TM} 1200 and 500 LIZ^{TM}; y axis – fluorescence intensity (peak height)

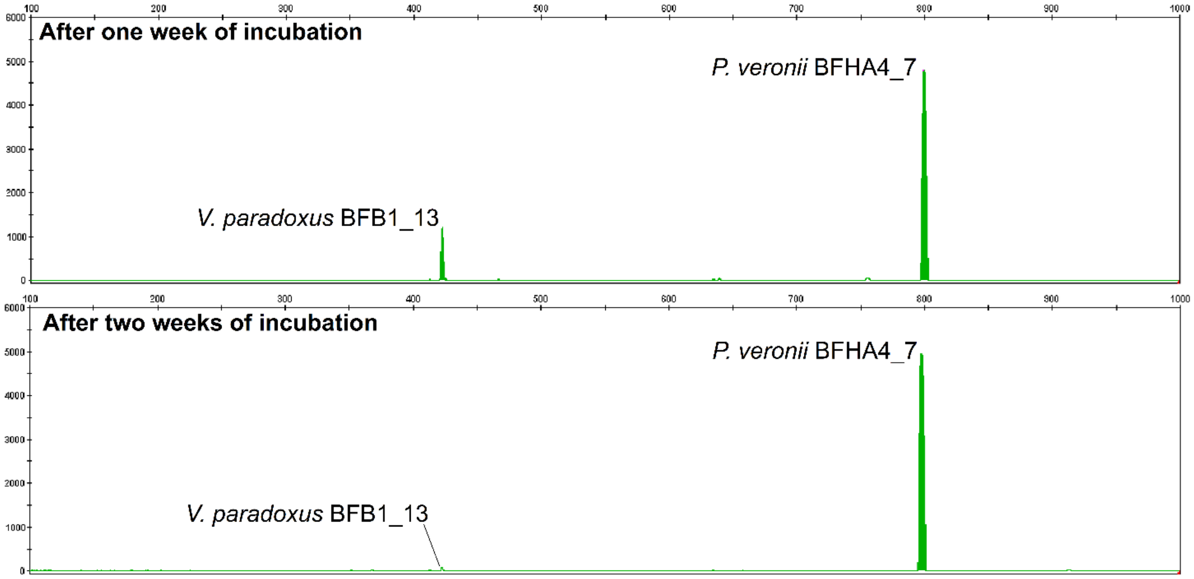

Fig. S5. Representative T-RFLP electropherograms obtained from co-cultured strains in a nutrient rich medium after one and two weeks of incubation. x axis – size of T-RFs (bp) according to the internal size standard GeneScan™ 1200 LIZTM; y axis – fluorescence intensity (peak height). RsaI restriction endonuclease was used for the generation of T-RFs

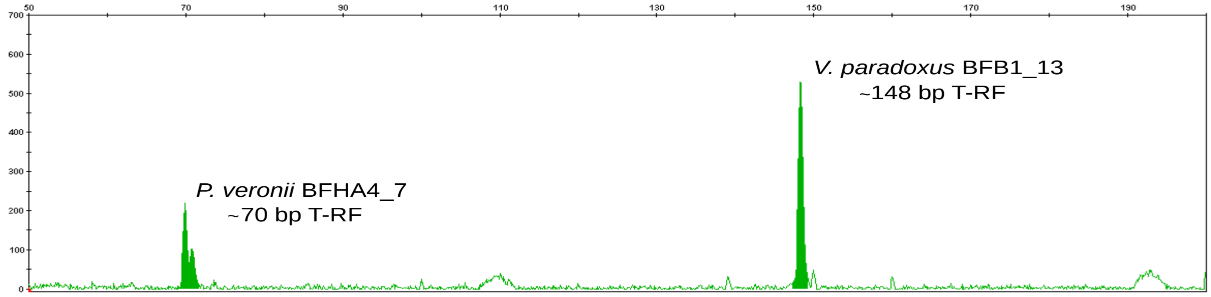

Fig. S6. A representative T-RFLP electropherogram obtained from co-cultured strains incubated for 8 days in mineral salts solution supplemented with BTEX compounds. x axis – size of T-RFs (bp) according to the internal size standard GeneScanTM 500 LIZ™; y axis – fluorescence intensity (peak height). AluI restriction endonuclease was used for the generation of T-RFLP profiles

Table S1: The mean concentration of BTEX and standard deviation values (STD) measured during testing biodegradation capacity of co-cultivated *V. paradoxus* BFB1_13 and *P. veronii* BFHA4_7 in mineral salts solution (Suppl. – supplementation with BTEX; h – hour).

**BTEX (mg l^{-1})**	**T0**	**STD**	**T0'**	**STD**	**T24**	**STD**	**T48**	**STD**
Benzene	1.66	0.11	1.31	0.04	0.47	0.10	0	0
Toluene	1.66	0.12	1.43	0.02	0.02	0.01	0	0
Ethylbenzene	1.66	0.12	1.46	0	1.24	0.05	0	0
o-xylene	1.66	0.12	1.50	0.03	0.99	0.07	0	0
m-xylene	1.66	0.14	1.60	0	0	0	0	0
p-xylene	1.66	0.09	1.50	0.22	0	0	0	0
**BTEX (mg l^{-1})**	**I. Suppl. (10 mg/l)**	**STD**	**T51 (I. Suppl. +3 h)**	**STD**	**T54 (I. Suppl. + 6 h)**	**STD**	**II. Suppl. (20 mg/l)**	**STD**
Benzene	1.66	0.11	1.32	0.24	0	0	3.30	0
Toluene	1.66	0.12	0.90	0.17	0	0	3.30	0
Ethylbenzene	1.66	0.12	1.65	0.15	0	0	3.30	0
o-xylene	1.66	0.12	1.54	0.15	0	0	3.30	0
m-xylene	1.66	0.14	0.16	0.07	0	0	3.30	0
p-xylene	1.66	0.09	0.10	0.05	0	0	3.30	0
**BTEX (mg l^{-1})**	**T69 (II. Suppl. +15 h)**	**STD**	**III. Suppl. (I. aeration - 60 ml; 20 mg/L)**	**STD**	**T75 (III. Suppl. + 6 h)**	**STD**	**IV. Suppl. (20 mg/L)**	**STD**
Benzene	0.01	0	3.30	0	0	0	3.30	0
Toluene	0.01	0	3.30	0	0	0	3.30	0
Ethylbenzene	0.01	0	3.30	0	0.01	0	3.30	0
o-xylene	0.02	0	3.30	0	0	0	3.30	0
m-xylene	0.01	0	3.30	0	0	0	3.30	0
p-xylene	0.01	0	3.30	0	0	0	3.30	0
**BTEX (mg l^{-1})**	**T104 (IV. Suppl. + 39 h)**	**STD**	**V. Suppl. (20 mg/L)**	**STD**	**T114 (V. Suppl. + 10 h)**	**STD**	**T128 (V. Suppl. + 24 h)**	**STD**
Benzene	0	0	3.30	0	0.08	0.03	0	0
Toluene	0	0	3.30	0	0	0	0	0
Ethylbenzene	0	0	3.30	0	0.58	0.22	0	0
o-xylene	0	0	3.30	0	0.25	0.18	0	0
m-xylene	0	0	3.30	0	0	0	0	0
p-xylene	0	0	3.30	0	0	0	0	0
**BTEX (mg l^{-1})**	**VI. Suppl. (II. aeration - 60 ml; 20 mg/L)**	**STD**	**T138 (VI. Suppl. + 10 h)**	**STD**	**T152 (VI. Suppl. + 24 h; III. aeration - 120 ml)**	**STD**	**T161 (VI. Suppl. + 33h; III. aeration + 9h)**	**STD**
Benzene	3.30	0	0.87	0.07	0.78	0.37	0.55	0.11
Toluene	3.30	0	0.04	0.01	0.08	0.08	0.03	0.01
Ethylbenzene	3.30	0	1.58	0.18	1.59	0.67	1.21	0.17
o-xylene	3.30	0	1.44	0.07	1.39	0.53	0.98	0.06
m-xylene	3.30	0	0	0	0	0	0	0
p-xylene	3.30	0	0	0	0	0	0	0
**BTEX (mg l^{-1})**	**T185 (VI. Suppl + 57 h; III. aeration + 33 h)**	**STD**						
Benzene	0	0						
Toluene	0	0						
Ethylbenzene	0	0						
o-xylene	0	0						
m-xylene	0	0						
p-xylene	0	0						

Table S2: The mean concentration of BTEX and standard deviation values (STD) measured during testing biodegradation capacity of the autochthonous bacterial community and of the inoculated groundwater microcosms.

				**Benzene (mg l^{-1})**				
	**T18**	STD	**T25**	STD	**T46**	STD	**T115**	STD
**Autochthonous Community**	0.30	0.05	0.26	0.02	0.20	0.06	0.00	0.00
**Autochthonous Community+ Inoculum**	0.37	0.07	0.22	0.01	0.23	0.06	0.00	0.00
				**Toluene (mg l^{-1})**				
	**T18**	STD	**T25**	STD	**T46**	STD	**T115**	STD
**Autochthonous Community**	0.30	0.03	0.30	0.04	0.22	0.04	0.07	0.01
**Autochthonous Community+ Inoculum**	0.28	0.06	0.13	0.00	0.13	0.03	0.00	0.00
				**Ethylbenzene (mg l^{-1})**				
	**T18**	STD	**T25**	STD	**T46**	STD	**T115**	STD
**Autochthonous Community**	0.49	0.04	0.48	0.00	0.28	0.05	0.00	0.00
**Autochthonous Community+ Inoculum**	0.51	0.08	0.43	0.02	0.39	0.09	0.00	0.00
				***o*-xylene (mg l^{-1})**				
	**T18**	STD	**T25**	STD	**T46**	STD	**T115**	STD
**Autochthonous Community**	0.34	0.02	0.34	0.00	0.27	0.04	0.23	0.01
**Autochthonous Community+ Inoculum**	0.35	0.04	0.31	0.02	0.31	0.05	0.00	0.00
				***m*-xylene (mg l^{-1})**				
	**T18**	STD	**T25**	STD	**T46**	STD	**T115**	STD
**Autochthonous Community**	0.40	0.04	0.41	0.02	0.31	0.02	0.32	0.02
**Autochthonous Community+ Inoculum**	0.27	0.10	0.05	0.02	0.03	0.01	0.00	0.00
				***p*-xylene (mg l^{-1})**				
	**T18**	STD	**T25**	STD	**T46**	STD	**T115**	STD
**Autochthonous Community**	0.44	0.04	0.42	0.02	0.33	0.07	0.31	0.02
**Autochthonous Community+ Inoculum**	0.22	0.03	0.00	0.00	0.00	0.00	0.00	0.00
